# Correction for Thompson et al., “High-Resolution Identification of Multiple *Salmonella* Serovars in a Single Sample by Using CRISPR-SeroSeq”

**DOI:** 10.1128/aem.01226-25

**Published:** 2025-09-18

**Authors:** Cameron P. Thompson, Alexandra N. Doak, Naufa Amirani, Erin A. Schroeder, Justin Wright, Subhashinie Kariyawasam, Regina Lamendella, Nikki W. Shariat

## AUTHOR CORRECTION

Volume 84, no. 21, e01859-18, 2018, https://doi.org/10.1128/aem.01859-18. Page 11: The following should be added to Materials and Methods.

‘Figure 3 was generated using the Excel-based CRISPR macro (P. Horvath, D. A. Romero, A.-C. Coûté-Monvoisin, M. Richards, H. Deveau, et al., J. Bacteriol. 190:1401–1412, 2008, https://doi.org/10.1128/jb.01415-07) that visually depicts the spacer content of each CRISPR array. The macro presents unique spacer sequences as different colored boxes. Spacers from the full-length arrays were copied from CRISPRFinder and pasted into the macro. Spacers from the CRISPR-SeroSeq output were pasted individually to align with the full-length array. Labels (sample IDs, “PCR” or “CSS” and “CRISPR1” and “CRISPR2”) were added in Excel, the image was copied into a PowerPoint slide, and the figure title was added.

For the serovar population analysis, the arrays were not assembled. Following BLAST alignment of sequencing reads to the CRISPR spacer database, spacers with a perfect match (typically 60.2 for a 32 bp spacer sequence) were retained and used in the analysis. Spacers with fewer than 10 reads were not included in the analysis, and serovars were only called if they contained multiple spacers that were unique to that serovar in our database.’

The authors would like to apologize for any confusion caused.

